# Aryl hydrocarbon receptor dependent anti-inflammation and neuroprotective effects of tryptophan metabolites on retinal ischemia/reperfusion injury

**DOI:** 10.1038/s41419-023-05616-3

**Published:** 2023-02-08

**Authors:** Yijie Yang, Ning Wang, Li Xu, Yixin Liu, Lulu Huang, Mengyang Gu, Yue Wu, Wenyi Guo, Hao Sun

**Affiliations:** 1 Department of Ophthalmology, Shanghai 9th People’s Hospital Affiliated to Shanghai Jiaotong University School of Medicine, Shanghai, China; 2 Shanghai Key Laboratory of Orbital Diseases and Ocular Oncology, Shanghai, China

**Keywords:** Neurodegeneration, Neuroimmunology

## Abstract

Glaucoma is the major cause of irreversible blindness in the world characterized by progressive retinal neurodegeneration, in which local inflammation in retina is involved in persistent loss of retinal ganglion cells (RGCs). In order to explore whether aryl hydrocarbon receptor (AhR) and its agonists tryptophan metabolites are involved in the development of glaucoma, we collected serum and retinas from non-glaucoma controls and patients with glaucoma. Results showed altered serum tryptophan metabolism and reduced retinal AhR expression in glaucoma patients. We also showed intraperitoneally injection of tryptophan metabolite 2-(1′H-indole-3′-carbonyl)-thiazole-4-carboxylic acid methyl ester (ITE) down-regulated retinal local inflammation and protected RGC apoptosis from retinal ischemia/reperfusion (IR) injury via AhR activation. We further revealed that ITE could inhibit inflammation in BV2 microglia and alleviate the neurotoxicity of microglial conditioned medium to RGCs under IR. Finally, we illustrated the possible mechanism that ITE limited ERK and NFκB dependent microglial inflammation. In summary, these findings suggest the critical role of tryptophan metabolism and retinal AhR signaling in modulating local inflammation mediated by microglia in glaucoma, and provide a novel avenue to targeting the intrinsically altered AhR signaling resulted from disturbed tryptophan metabolism for glaucoma treatment.

## Introduction

Glaucoma is the leading cause of irreversible visual loss around the world. Elevation of the intraocular pressure (IOP) is the major risk factor of glaucoma, resulting in a series of pathophysiological changes of the retina and finally causing loss of retinal ganglion cell (RGC) and reduced visual field [1]. Intervention of IOP is the consensus in glaucoma treatment. However, it cannot always meet the goal to prevent the progress of visual loss even if the major risk factor IOP is well controlled [2]. Besides, retinal local inflammation mediated by glial cells is considered to be another impactful etiology of neurodegeneration in glaucoma [3]. Although the adaptive roles are beneficial in early stage of tissue repair, glial cells confer prolonged cytotoxicity to RGCs through amplified inflammation [3, 4]. Substantial studies have identified various sensors, transducers and cytokines linking the neuroinflammation of glial cells to RGC loss, but there are no drugs targeting glial cells used in clinical treatment of glaucoma currently [4–7]. At present, some synthetic molecules mainly about inhibition the activation of glial cells with known anti-inflammatory effects or antagonists of inflammatory sensors, transducers and receptors on glial cells, are being studied for therapeutic effect on limiting retinal inflammation and reducing RGC loss [4, 8, 9]. However, the regulation of intrinsic changes that associated with local inflammation during glaucoma remains unclear. Treatment strategies aimed to intrinsically regulate the dysregulated local inflammation in glaucoma may achieve unexpected and satisfying results on the basis of IOP reduction therapy.

Recently, tryptophan metabolism has been demonstrated to be associated with immune and inflammation in central nerves system (CNS) [10, 11]. Tryptophan is an essential amino taken from diets, which can be metabolized by the host and the gut microbiota. The host accounts for more than 90% of tryptophan metabolism via two major pathways, the kynurenine pathway and the serotonin pathway [12]. A small part of tryptophan will be metabolized through the indole pathway mediated by gut microbiota. Many effective metabolites such as 2-(1’H-indole-3’-carbonyl)-thiazole-4-carboxylic acid methyl ester (ITE) are also important products of tryptophan metabolism [13, 14]. Since first discovery of their immunosuppressive effect [15], tryptophan metabolites have attracted much attention in the field of neurodegenerative diseases, including multiple sclerosis (MS) [16]. The level of tryptophan microbial metabolites and kynurenines in serum and cerebrospinal fluid is significantly reduced in patients with MS [10, 17, 18]. In a commonly used murine model of MS, supplementation of tryptophan microbial metabolites could obviously alleviate CNS inflammation through activation of aryl hydrocarbon receptor (AhR) [10]. AhR was first known as a receptor of environmental pollutants such as polycyclic and halogenic aromatic hydrocarbons [19]. The immune-regulatory potential of AhR responding to endogenous tryptophan metabolites to exert anti-inflammatory effects has been recently shown in various diseases [20–23]. AhR is a ligand-activated transcription factor that belongs to the PER–ARNT–SIM superfamily [20]. When bound with ligands like tryptophan metabolites, AhRs translocate into the nucleus with the combination of Ah receptor nuclear translocator (ARNT), bind to the AhR-responsive DNA elements and regulate the transcription of targeted genes [20]. Moreover, Nuclear factor-κB (NFκB) has been reported to be one of the targeted factors of AhR in CNS, which is a key transcriptional activator in inducing glial cells mediated inflammation in neurodegenerative diseases [24, 25]. Activation of AhR significantly inhibited NFκB signaling, reduced levels of downstream inflammatory cytokines like TNF-α, IL6 and CCL2 and relieved CNS inflammation in MS models, while AhR deletion resulted in increased nuclear localization of NFκB in microglia [10, 11]. As an important part of nervous system, retina is considered to be an extent of the brain. However, the role of tryptophan metabolism in retinal neurodegeneration in glaucoma remains unexplored. Of note, although reduced level of kynurenine in retina and serum has been found in hereditary glaucoma model [26], the overall tryptophan metabolism signature in glaucoma and the effects of tryptophan metabolism on retinal neurodegeneration have not been elucidated. Furthermore, whether AhR in microglia is involved in the regulation of retinal local inflammation warrants further exploration.

Here, we collected serum and retina samples from glaucoma patients and non-glaucoma controls and detected significant decrease of serum tryptophan metabolites and reduced retinal AhR expression in glaucoma patients. We also showed that supplementation of tryptophan metabolite ITE could inhibit retinal inflammation and alleviate retinal injury in IR mice and limit inflammation in BV2 microglia cell via AhR activation. Moreover, cytokine antibody array revealed that ITE could significantly decrease the pro-inflammatory cytokines induced by LPS, while increase the anti-inflammatory cytokine IL-10, thus reduce the neurotoxicity of BV2 conditioned medium to RGCs under IR. Finally, we revealed AhR activation by ITE could down-regulate NFκB and ERK pathways in microglia to confer anti-inflammation effect. Taken together, these findings establish a critical link between perturbed tryptophan metabolism and dysregulated inflammation mediated by reduced AhR activation of microglia in glaucoma. We also highlight that supplementation of relative endogenous metabolites aiming to refine intrinsic tryptophan metabolism and AhR signaling may be a more effective strategy to limit amplified inflammation associating local glial cells for glaucoma treatment.

## Results

### Altered tryptophan metabolism and AhR signaling was found in glaucoma patients

**Fig. 1:**
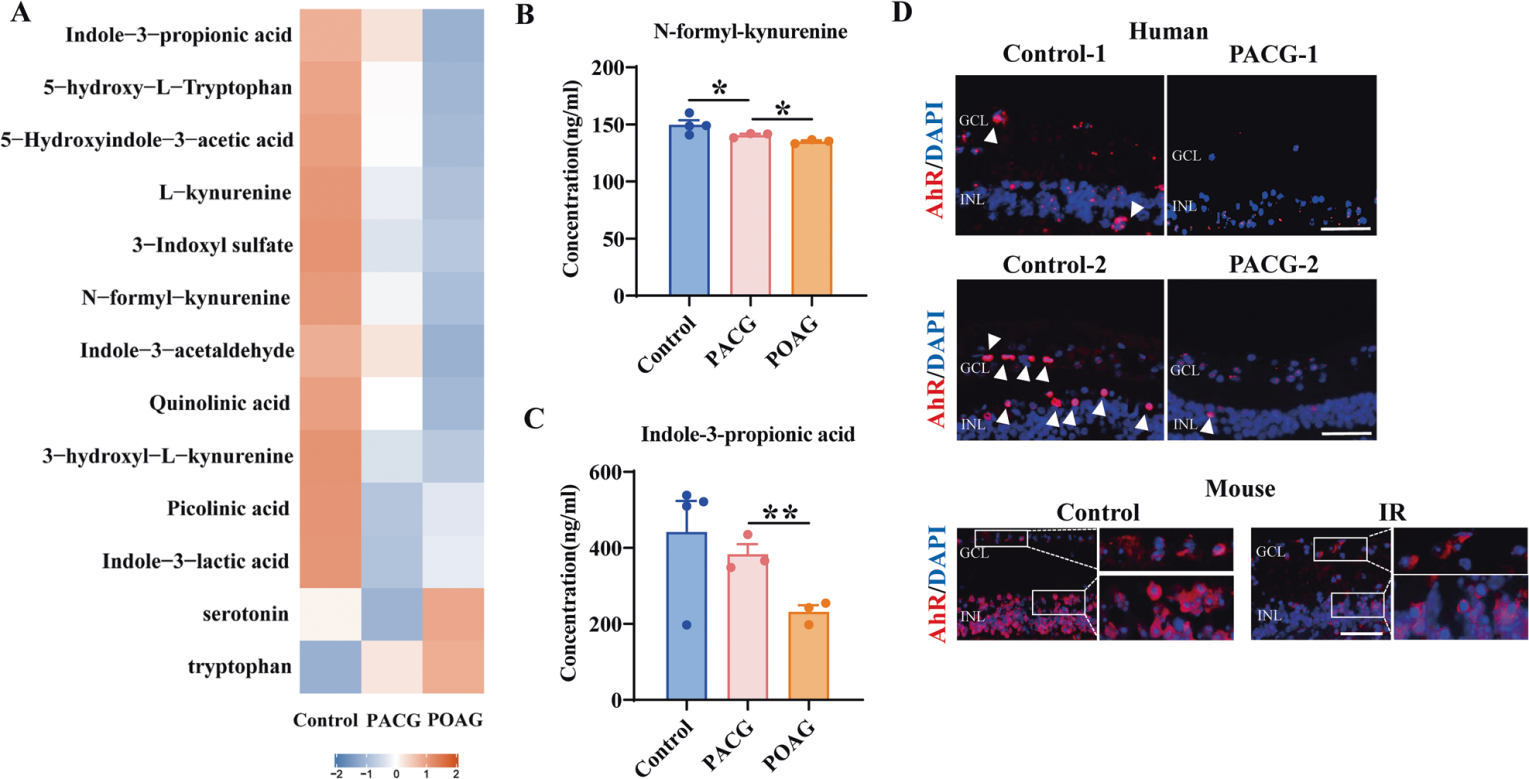
Tryptophan metabolites and retinal AhR were abnormally expressed in glaucoma patients. **A** Metabolomics of serum from control (*n* = 4), PACG (*n* = 3) and POAG (*n* = 3) patients to detect the differential tryptophan and tryptophan metabolites expression signatures. **B**, **C** Histogram showing concentration of N-formyl-kynurenine and indole-3-propionic acid of three groups. **D** Immunofluorescence staining showed reduced expression of AhR (red) in human retinal sections from PACG patients (*n* = 2) than that of Control group (*n* = 2), and in mice retinal sections from IR injury at day 1 (*n* = 3) than controls (*n* = 3). Cell nuclei were counterstained with DAPI (blue). GCL ganglion cell layer, INL inner nuclear layer. Scale bar = 50 µm. *P < 0.05, **P < 0.01, *P* value was obtained by one way ANOVA.

As AhR ligands, tryptophan metabolites play important roles in immune and inflammation regulation [20]. We first sought to investigate whether tryptophan metabolism participate in the development of glaucoma, and therefore targeted metabolomics of serum from patients with primary open angle glaucoma (POAG), primary angle-closure glaucoma (PACG) and non-glaucoma controls were performed. There was a significant decrease of serum tryptophan metabolites in both types of glaucoma compared with controls and even lower level in POAG patients compared with PACG patients (Fig. 1A). N-formyl-kynurenine and Indole-3-propionic acid, the representative metabolites of kynurenine pathway and indole pathway respectively, were significantly reduced in POAG (Fig. 1B, C). Interestingly, the level of tryptophan and serotonin were higher in POAG patients than other groups (Fig. 1A). The accumulation of tryptophan in POAG patients may be due to reduced catabolism itself through indole and kynurenine pathways, which also caused perturbed serotonin pathway and increased level of serotonin. Notably, tryptophan is an important source of AhR agonists that can be metabolized into AhR ligands mainly via indole and kynurenine pathways, but not the serotonin pathway [22]. This means a significant reduce of AhR ligands generated by tryptophan in glaucoma, especially POAG patients. Therefore, we compared the retinal AhR expression of PACG patients and non-glaucoma controls, given that the great difficulty of acquisition of eyeball samples from POAG patients. Immunofluorescence staining of eyeball sections showed that AhR were spot-shaped distributed in the inner retina in normal controls. Expectedly, reduced AhR expression was found in the inner retina with PACG compared with controls (Fig. 1D). Similar findings were also showed in mice IR injury, a well-validated model of glaucoma [27, 28] (Fig. 1D). The impaired AhR signaling in patients with PACG indicated that immune and inflammatory factors may be also involved in PACG, which is consistent with other studies that revealed high level of inflammatory cytokines both locally and systemically in PACG [29, 30]. Taken together, these findings indicated that altered tryptophan metabolism and reduced tryptophan derived AhR ligands may cause insufficient retinal AhR activation in glaucoma. We next investigated whether AhR signaling was involved in the development of glaucoma.

### Tryptophan metabolite ITE alleviated retinal injury and ameliorated retinal local inflammation in IR mice model via AhR activation

**Fig. 2:**
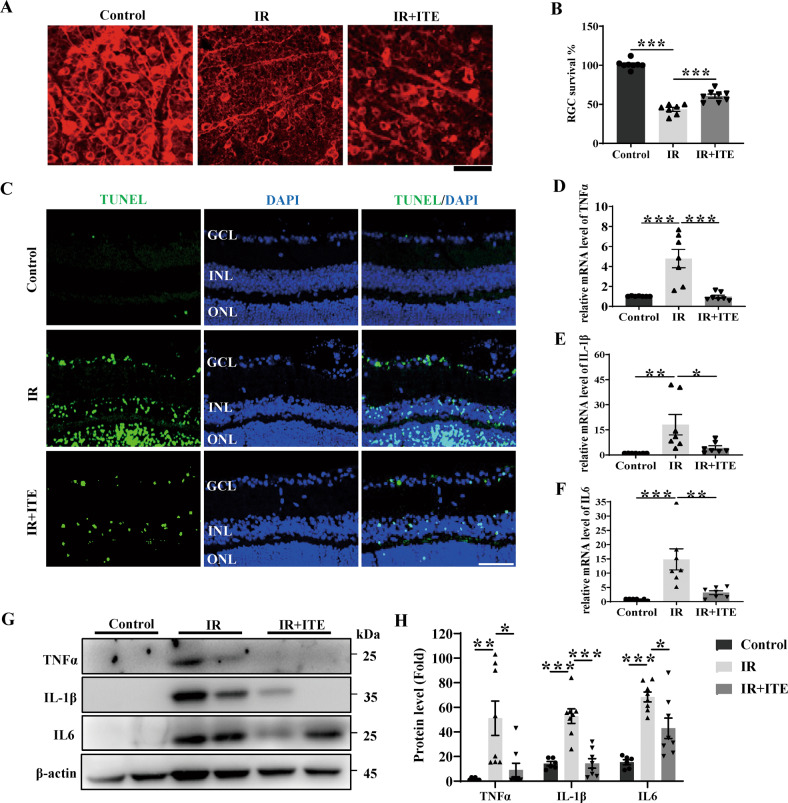
Tryptophan metabolite ITE alleviated retinal injury and ameliorated retinal inflammation state in IR mice model. Retinal ischemia/reperfusion injury model was established with or without ITE (10 mg/kg) daily intraperitoneal injection, and mice were euthanatized after 7 days or 1 day for RGC survival investigation or 1 day to detect the expression of inflammatory cytokines. **A** Retinal flat mounts from Control, IR and IR + ITE groups at day 7 to detect β-III-tubulin (red) positive RGCs by immunofluorescence staining. **B** Histograms showing quantitation of RGC survival percent in each group. **C** Apoptosis of the retina from Control, IR and IR + ITE groups at day 1 were detected by TUNEL (green) staining. Cell nuclei were counterstained with DAPI (blue). GCL ganglion cell layer, INL inner nuclear layer, ONL outer nuclear layer. **D**–**F** mRNA expression of TNFα, IL-1β and IL6 at day 1 were detected by qRT-PCR in the retina from Control, IR and IR + ITE groups. **G**, **H** Western blot showing the protein expression of inflammatory cytokines TNFα, IL-1β and IL6 in retinas of IR at day 1 with or without ITE administration. Control, control group; IR, ischemia/reperfusion injury; IR + ITE, IR with ITE treatment. A-B, D-H, *n* *=* *6-8* for each group; C, *n* = 4 for each group, scale bars: 50 μm. All data are presented as mean ± SEM, *P < 0.05, **P < 0.01, ***P < 0.001, *P* value was obtained by one way ANOVA followed by multiple comparisons. Each dot in graphs represents data from an individual retina from mice.

ITE is an endogenous AhR agonist generated from tryptophan metabolism [14]. Because of its non-toxic characteristic, ITE has been considered as a benchmark AhR endogenous agonist in various studies [31]. Here, we intraperitoneally injected ITE for mice at the same time with induction of retinal IR injury. At day 7, mice retinal-mount with RGC labeling showed that ITE could significantly alleviate the RGC loss caused by IR (Fig. 2A). As shown in Fig. 2B, a significant reduce of RGC number to 43.6% was observed after IR, which was improved to 60.6% with ITE treatment. Moreover, TUNEL staining showed that ITE also significantly reduced RGC apoptosis induced by IR at day 1 (Fig. 2C). To examine how ITE could reduce RGC loss during IR, we next explored the retinal inflammation status in mice. Due to the early change of retina inflammatory factors, the mRNA level of TNF-α, IL-1β and IL6, which are the most frequently induced cytokines during inflammation, were found to be significantly up-regulated at 24 h after IR, while significantly down-regulated with ITE administration (Fig. 2D–F). Similarly, treatment with ITE significantly reduced the induction of TNF-α, IL-1β and IL6 at protein level (Fig. 2G, H). These data indicated ITE could alleviate RGC injury under IR through inhibiting retinal local inflammation.

**Fig. 3:**
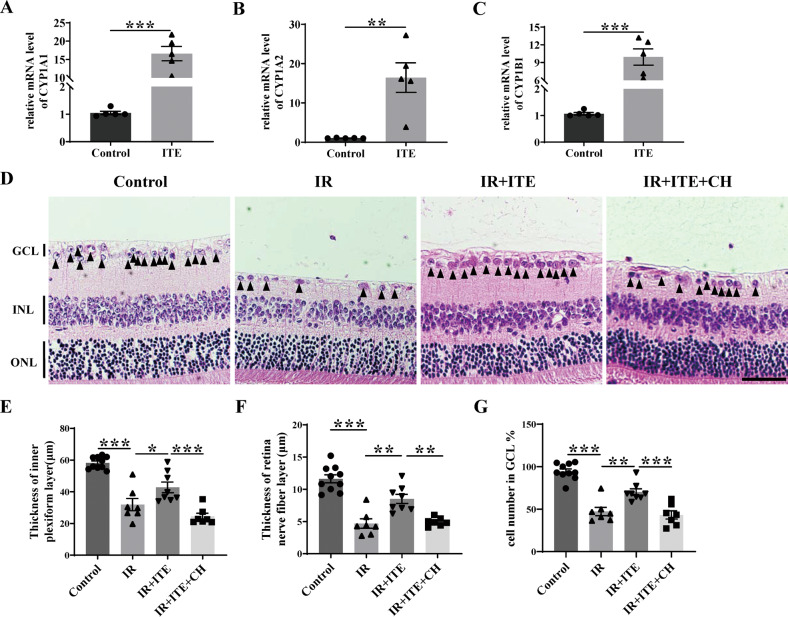
The protective role of ITE in alleviating retinal injury was AhR activation dependent. Retinal ischemia/reperfusion injury model mice were intraperitoneally injected with ITE or CH223191 (10 mg/kg, inhibitor of AhR and abbreviated as CH below). **A**–**C** mRNA expression of CYP1A1, CYP1A2, and CYP1B1 were detected by qRT-PCR in the retina of mice with or without ITE administration for 24 h. **D** H&E staining showed different histology of retinas 7 days after IR. GCL ganglion cell layer, INL inner nuclear layer, ONL outer nuclear layer. Scale bars: 50 μm. **E**–**G** Quantitative analysis of the thickness of inner plexiform layer and retina fiber layer and cell number of ganglion cell layer were shown in histograms. Control, control group; ITE, ITE treatment; IR, ischemia/reperfusion injury; IR + ITE, IR with ITE treatment; IR + ITE + CH, IR with ITE and CH223191 treatment, *n* = 5–10 for each group. All data are presented as mean ± SEM, *P < 0.05, **P < 0.01, ***P < 0.001, *P* value was obtained either by Student’s t test or by one way ANOVA followed by multiple comparisons. Each dot in graphs represents data from an individual retina from mice.

Next, we further investigated whether AhR activation was required for the protective role of ITE. As shown in Fig. 3A–C, intraperitoneal injection of ITE significantly enhanced the mRNA level of AhR directly targeted genes CYP1A1, CYP1A2 and CYP1B1, indicating that the effect of ITE could cross through the blood-retinal barrier (BRB) and activate retinal AhR after treatment for 24 hours. Further, the AhR antagonist CH223191 was used to inhibit AhR activation [32], and differential morphological changes were observed in retinal sections (Fig. 3D). The thickness of inner plexiform layer (IPL) and retinal nerve fiber layer (RNFL) were significantly decreased after IR, while ITE supplementation markedly relieved the structural thinning (Fig. 3E, F). In addition, ITE effectively attenuated cell loss in ganglion cell layer (GCL) caused by IR (Fig. 3G). Importantly, these protective effects of ITE were dampened after CH223191 treatment (Fig. 3D–G), confirming that the protective role of ITE was AhR dependent.

### Tryptophan metabolite ITE limited LPS induced inflammation in BV2 microglia via AhR activation

**Fig. 4:**
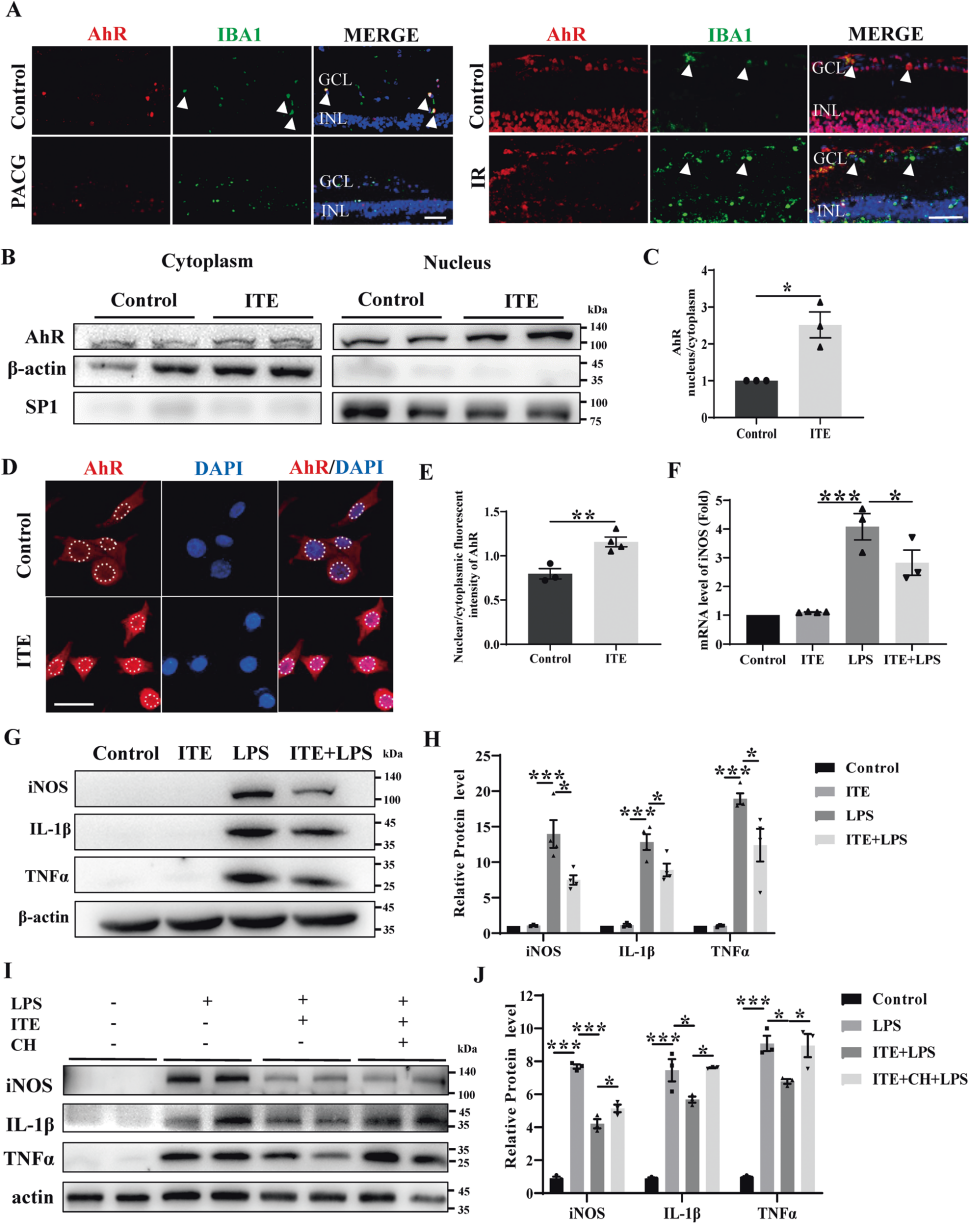
ITE alleviated LPS induced inflammation via activating AhR in BV2 microglia. **A** Immunofluorescence staining showed co-location of AhR (red) and IBA1 (green) in human and mice retinal sections from control group, while decreased retinal AhR expression in PACG patients and mice IR injury model (at day 1). Cell nuclei were counterstained with DAPI (blue). GCL ganglion cell layer, INL inner nuclear layer. Scale bar = 50 µm. **B**, **C** BV2 microglia was treated with ITE (1 μM) for 4 h. Subcellular fractionation was performed, SP1 and β-actin were served as loading control for nucleus and cytoplasm respectively. Representative western blot analysis for AhR **B** and quantification of the ratio of nuclear to cytoplasmic fraction **C**. **D** Immunofluorescence staining for AhR (red) to show its nuclear translocation after ITE treatment for 4 h compared with Control. Cell nuclei were counterstained with DAPI (blue). Scale bar: 25 μm. **E** Quantification of nuclear/cytoplasmic fluorescent intensity of AhR in Control and ITE groups. **F** BV2 cells were pre-treated with ITE for 4 h and followed by LPS (100 ng/ml) for 20 h. mRNA level of iNOS was detected by qRT-PCR in BV2 microglia of Control, ITE, LPS and ITE + LPS groups. **G**, **H** Western blot of each group showing protein expression of iNOS, IL-1β and TNFα in BV2 microglia. **I**, **J** CH223191 was added 24 h before ITE treatment. Western blot of each group showing the protein level of iNOS, IL-1β and TNFα in BV2 microglia. All data are presented as mean ± SEM. and are representative of at least three independent experiments. *P < 0.05, **P < 0.01, ***P < 0.001, *P* value was obtained either by Student’s t test or by one way ANOVA followed by multiple comparisons.

Given that microglia play the most important role in initiating and amplifying inflammatory process during glaucoma [3], and previous studies have shown that microglial AhR play vital roles in limiting CNS inflammation [11], we next demonstrated the cellular localization of retinal AhR in human and mice sample. AhR was found to be co-localized with IBA1, the marker of microglia in controls, but not obvious in PACG (Fig. 4A), which may be due to the decrease of retinal AhR expression in PACG. In similar, co-localization of IBA1 and AhR was more demonstrable in controls compared with IR mice retina (Fig. 4A). We also harnessed BV2 microglia cell to explore the mechanisms by which AhR activation can protect against IR. In order to assess the efficacy on AhR activation of ITE in BV2 microglia, which to our knowledge has not been tested yet by others, we first examined the nucleus translocation of AhR. A significant increase of nucleus/cytoplasm AhR was found after ITE treatment either by nuclear-cytoplasm-protein western blotting (Fig. 4B, C) or immunofluorescence staining (Fig. 4D, E) in BV2 microglia, indicating the activation of microglial AhR by ITE. Consistent with the results in vivo, ITE supplementation resulted in a significant attenuation of the LPS mediated induction of TNF-α, IL-1β and iNOS in BV2 microglia (Fig. 4F–H). However, the anti-inflammatory effect of ITE was obviously weakened with the administration of AhR antagonist CH223191, indicating that inflammation inhibition of ITE was AhR dependent in BV2 microglia (Fig. 4I, J).

### ITE limited the neurotoxicity of microglia conditioned medium to mice retina under IR

**Fig. 5:**
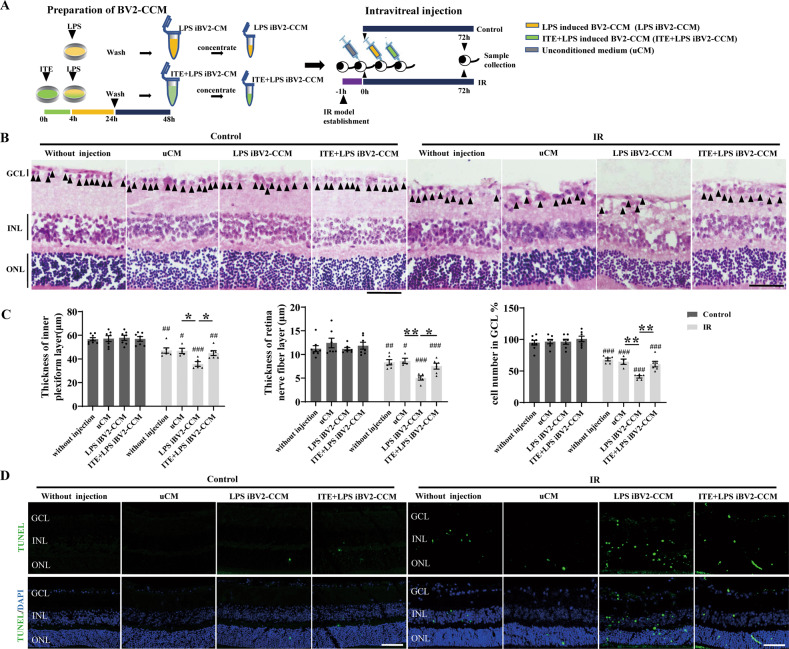
ITE conferred retinal protection against microglia derived toxins induced by LPS in retinal IR injury. **A** Experimental protocols. LPS was added for 20 h with or without ITE pre-treatment for 4 h. BV2 microglia were then washed with PBS and medium was changed. After 24 hours, BV-2 conditioned medium was collected and concentrated as LPS induced BV2-CCM or ITE + LPS induced BV2-CCM. Mice were subjected to IR one hour before intravitreal injection with relative medium. Mice were euthanatized at day 3 after IR injury. **B** H&E staining of frozen tissue sections showed retinas in Control or IR groups receiving different conditioned medium. GCL ganglion cell layer, IPL inner plexiform layer, INL inner nuclear layer, ONL outer nuclear layer. Scale bar = 50 µm. **C** Quantitative analysis of the thickness of inner plexiform layer and retinal nerve fiber layer and cell numbers in ganglion cell layer in each group. **D** Apoptosis of the retina from Control and IR groups were detected by TUNEL (green) staining. Cell nuclei were counterstained with DAPI (blue). Scale bar = 50 µm. All data are presented as mean ± SEM, and are representative of 4-8 independent experiments. *P < 0.05, **P < 0.01. Compared with Control group: ^#^*P* < 0.05, ^##^*P* < 0.01, ^###^*P* < 0.001. *P* value was obtained either by Student’s t test or by one way ANOVA followed by multiple comparisons.

In order to explore the effect of microglial AhR activation on regulating retinal injury, we induced inflammation of BV2 cells by LPS, with or without ITE pre-treatment, and collected BV2 concentrated conditioned medium (BV2-CCM) to intravitreally inject to mice, aiming to detect the effect of microglia derived cytokines regulated by AhR on retinal injury. Experimental protocols were summarized in Fig. 5A. Due to the severe injury at day 7 after IR, we collected retinas at day 3 in order to observe the effects of BV2-CCM on mice retinas. Expectedly, in mice without IR injury, LPS induced BV2-CCM injection had no effect on the retinal thickness or cell number of GCL, indicating that RGC pre-injury is an essential prerequisite for the susceptibility to toxins. However, completely different results were shown under IR injury (Fig. 5B, C). LPS-induced BV2-CCM (IR-LPS iBV2-CCM group) significantly aggravated the retinal injury, manifested by the further thinning of the IPL and RNFL, as well as the more decreased cell number of GCL than unconditioned medium (IR-uCM group). These further injuries were not seen in IR mice when paired with injection of ITE + LPS induced BV2-CCM (Fig. 5B, C). Moreover, injection of LPS-induced BV2-CCM aggravated retinal apoptosis as shown by TUNEL staining in IR mice compared with unconditioned medium (IR-uCM group). This aggravation was not shown when injection of ITE + LPS-induced BV2-CCM (Fig. 5D). Notably, although injured retinas were susceptible to LPS induced microglial toxins, intravitreally injection of ITE + LPS-induced BV2-CCM would not cause further neurotoxicity to retinas under IR (Fig. 5B, D). These results indicated the possibility that activation of AhR by ITE could effectively relieve LPS-induced microglial neurotoxicity on IR retina via limiting the secretion of neurotoxins in microglia.

### ITE alleviated NFκB and ERK dependent inflammation via AhR activation in BV2 microglia

**Fig. 6:**
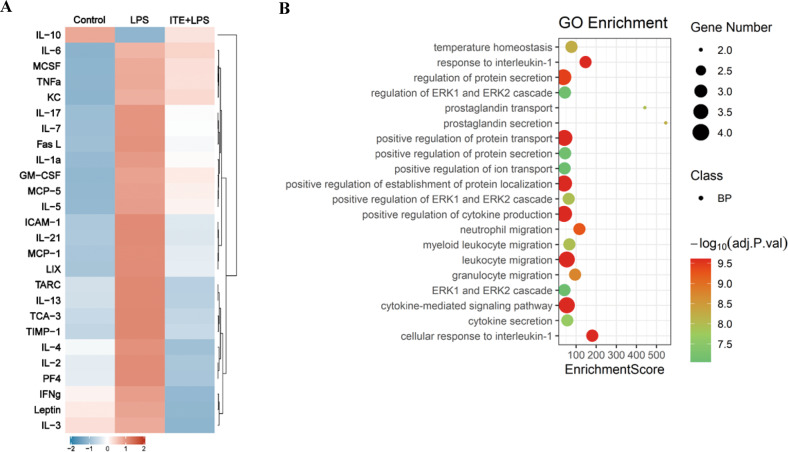
ITE extensively inhibited secretion of LPS induced inflammatory cytokines in BV2 microglia. **A** RayBio mouse inflammatory cytokine antibody array was applied to detect inflammatory cytokine signatures in BV2 microglia cell conditioned medium of three groups. Control, unconditioned BV2-CCM; LPS, LPS induced BV2-CCM; ITE + LPS, ITE + LPS induced BV2-CCM. Inflammatory cytokines were significantly reduced with ITE pre-administration. **B** GO biological process analysis of the top 20 processes involved in ITE + LPS vs LPS group. *n* = 3 in control group, *n* = 4 in LPS and ITE + LPS groups.

In order to verify the role of microglial AhR mentioned above and further elucidate the probable cytokines secreted from microglia, inflammatory cytokine antibody array was used to detect a panel of cytokines in BV2-CCM. As shown in Fig. 6A, LPS significantly induced a broadscale of the pro-inflammatory cytokines, among which inflammatory factors (such as TNFα, IL-1α and IL6) and chemokines (such as MCP-5, MCP-1 and TCA-3) were in the majority. ITE treatment strongly reduced the level of these cytokines. Importantly, the anti-inflammatory cytokine IL-10 was increased with ITE administration (Fig. 6A), indicating the role of ITE in maintaining the balance of pro- and anti-inflammation. To make it further, Gene ontology (GO) analysis was performed and top 20 biological processes that may be involved in microglia mediated inflammation were revealed (Fig. 6B). We found a predominant role of ITE in regulating some critical biological functions, including cytokine-mediated signaling pathway and positive regulation of cytokine production, which just explained the anti-inflammatory role of ITE (Fig. 6B). Notably, among all the biological processes, extracellular signal regulated kinase (ERK) cascade was also of great importance (Fig. 6B).

**Fig. 7:**
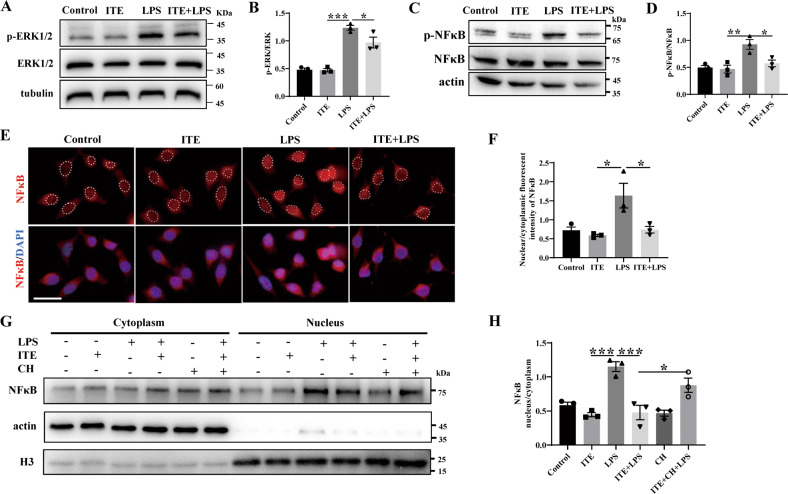
ITE alleviated LPS induced NFκB and ERK signaling via AhR activation in BV2 microglia. BV2 cells were pre-treated with ITE for 4 h and followed by LPS for 4 h. **A**, **B** Western blot showing the expression of p-ERK and ERK in BV2 microglia of Control, ITE, LPS and ITE + LPS groups. **C**, **D** Western blot of each group showing the protein level of p-NFκB and NFκB in BV2 microglia. **E** Immunofluorescence staining to detect different nuclear translocation state of NFκB (red) in each group. Cell nuclei were counterstained with DAPI (blue). Scale bar: 25 μm. **F** Quantification of nuclear/cytoplasmic fluorescent intensity of NFκB in four groups. **G**, **H** CH223191 was added 24 h before ITE treatment and subcellular fractionation separation experiments was performed. Representative western blot analysis for NFκB **G** and quantification of the ratio of nuclear to cytoplasmic fraction **H**. All data are presented as mean ± SEM. and are representative of at least three independent experiments. *P < 0.05, **P < 0.01, ***P < 0.001, *P* value was obtained by one way ANOVA followed by multiple comparisons.

We next detected the phosphorylation of ERK by western blot and demonstrated that ITE treatment significantly down-regulated LPS-induced phosphorylation of ERK in BV2 microglia (Fig. 7A, B). Given that NFκB is the key transcriptional factor that mediates the neuroinflammation process of glaucoma [3, 25], NFκB was also detected. As shown in Fig. 7C, D, ITE treatment reduced almost 75% of phosphorylation of NFκB induced by LPS. Immunofluorescence staining and nuclear-cytoplasm-protein western blotting were thus performed and revealed that ITE treatment significantly reduced LPS induced translocation of NFκB from cytoplasm to nucleus (Fig. 7E–H), confirming the important role of ITE in limiting NFκB activation. Moreover, these reverse effects of ITE were suppressed with the treatment of AhR antagonist CH223191 (Fig. 7G–H), supporting the AhR dependent role in ITE inhibiting the activation of NFκB. Taken together, these results indicated that AhR activation could down-regulate LPS-induced ERK and NFκB signaling in BV2 microglia and thus limit downstream inflammation.

**Fig. 8:**
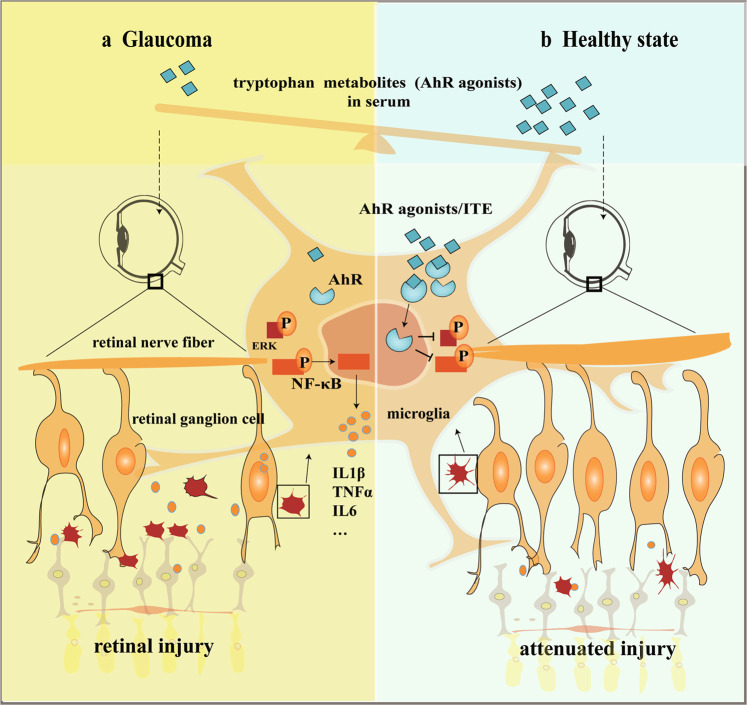
AhR dependent anti-inflammation and neuroprotective effects of tryptophan metabolites on retinas under ocular hypertension. Disturbed serum tryptophan metabolism and reduced AhR agonists are shown in glaucoma state, which result in reduced microglial AhR activation. Amplified local inflammation mediated by microglia, which are ERK and NFκB signaling dependent, could cause significant neurotoxicity to RGCs under ocular hypertension (**a**, left). However, tryptophan metabolism is normal and there are enough AhR agonists in healthy state. Supplementation of tryptophan metabolites such as ITE could significantly inhibited ERK and NFκB dependent local inflammation and attenuated retinal injury under ocular hypertension via AhR activation (**b**, right). In summary, AhR activation by tryptophan metabolites play anti-inflammation and neuroprotective roles in glaucoma.

In summary, our results illustrated the unbalanced tryptophan metabolism in glaucoma patients and the role of AhR signaling in retinal neuroprotection. Supplementation of tryptophan metabolite ITE could down-regulate ERK and NFκB dependent inflammation of microglia through AhR activation and thus alleviate retinal injury under IR in mice (Fig. 8), raising the possibility that intrinsic regulation of AhR signaling by supplementation of tryptophan metabolites can serve as a novel avenue for the treatment of glaucoma.

## Discussion

The prevalence of glaucoma is persistently increasing in recent years with aging of population [33]. It is noteworthy that progression of visual loss often exists even IOP is under control by current therapeutic methods. How to prevent irreversible visual field defect and blindness caused by glaucoma remain challenging, and new strategy besides controlling the IOP is warranted. In the current study, we propose that the development of glaucoma may involve the insufficiency of microglial AhR activation in retina, which may be resulted from altered tryptophan metabolism in glaucoma patients. We also shed light on the potential of supplementation of tryptophan metabolites like ITE to attenuate retina inflammation and RGC loss under IR through inhibiting ERK and NFκB pathway in microglia via AhR activation. Here the protective role of tryptophan metabolites/AhR pathway limiting dysregulated local inflammation mediated by microglia in glaucoma were studied in detail.

Tryptophan metabolic changes have been found in CNS neurodegenerative diseases, and the role of tryptophan metabolism in both diagnosis and treatment has attracted much attention in recent years [12, 34, 35]. Recently, tryptophan metabolism in glaucoma has been studied gradually. Pulukool et al. showed activated kynurenine pathway in POAG, which was associated with microglia-mediated inflammation [36], while kynurenine was shown to be decreased in serum and retina of a mice glaucoma model [26]. The different changes of metabolites may come from different tissues and the complex regulation of tryptophan metabolism, however, they all indicated the involving of perturbed tryptophan metabolism in glaucoma. Here we discovered that tryptophan metabolites, as a very important category of AhR ligands, like N-formyl-kynurenine and indole-3-propionic acid decreased significantly in the serum of POAG and PACG patients, suggesting that AhR associated inflammation may be involved in both POAG and PACG. Notably, the level of these tryptophan metabolites was even lower in patients with POAG than PACG, indicating that disturbed tryptophan metabolism and related inflammation may play more important roles in POAG. It is in line with widely accepted that neuroinflammation takes significant roles in the development of POAG, while increased IOP resulted from angle closure is considered to be the major pathogenic factor of PACG [37]. Targeted metabolomics of human serum tryptophan signature revealed AhR related pathways were down-regulated, represented by decreased N-formyl-kynurenine and indole-3-propionic for kynurenine and indole pathways respectively in glaucoma patients. While the up-regulated serotonin was also noted in glaucoma, which is not the widely reported AhR related pathway (Fig. 1A–C). We thus postulated the down-regulation of kynurenine and indole pathways resulted in accumulation of tryptophan in glaucoma patients, which further caused a compensatory up-regulation of serotonin pathway. In this study, we focused on the effect of reduced AhR related pathways on glaucomatous retina through local microglial inflammation, while further studies are needed to investigate if the increased serotonin pathway is also involved in the development of POAG. Of note, the influence of peripheral dysregulated tryptophan metabolism on retinal AhR indicated that there may be a possible axis linked peripheral metabolism and the retina, providing new evidence for proposed gut-retina axis mediated by microbiota [38, 39]. Since tryptophan can be metabolized by multiple pathways [40], the initial factor of disturbed tryptophan metabolism in patients with glaucoma needs further investigation.

Substantial endeavors have been done to seek neuroprotective therapy in glaucoma via modulating genetic and epigenetic susceptibility, mitochondrial dysfunction, glia mediated local inflammation, systemic dysregulation of T lymphocytes and microbiota and so on [3]. Here, we postulated a new perspective of supplementing endogenous tryptophan derived AhR ligands to confer neuroprotection in glaucoma. We aimed to modulate the intrinsic changes of tryptophan metabolism and AhR signaling that affect microglia activity to inhibit local inflammation in glaucoma [28, 41]. Previous study discussed the role of tryptophan metabolism in POAG, mainly focusing on the association of microglia mediated neurotoxicity [36]. However, it’s worth noting that microglia, on the one hand, involve in the pro-inflammatory process through producing neurotoxic metabolites and cytokines, on the other hand, also take part in the anti-inflammatory process through AhR activation [20]. It is possible that the balance of these two aspects were perturbed during glaucoma. Moreover, administration of more down-stream tryptophan metabolites may be more appropriate to confer neuroprotective effects, avoiding produce of neurotoxic metabolites by microglia. In our study, the strategy of activating AhR by the tryptophan metabolite ITE could alleviate retinal inflammation, delay retina thinning and RGC loss under IR (Figs. 2–3). Furthermore, AhR was shown to co-localize with microglia in human and mouse retina, and activation of microglia AhR could significantly limit ERK and NFκB mediated inflammation and reduce its toxicity to neurons under IR injury (Figs. 4–7). These results indicated the effective role of tryptophan metabolism in regulating the amplified local inflammation mediated by microglia in glaucoma through AhR, and provided theoretical basis for clinical transformation. Previous clinical research in glaucoma relevant to neuroprotection are mainly consist of drugs that are synthetic small molecules (DNB-001, QPI-1007, etc) or already used for other purposes (brimonidine, memantine, etc) [42, 43]. The 2-year phase 3 clinical trial of over 2,000 patients with open-angle glaucoma showed no effects to prevent glaucomatous progression by daily treatment with memantine [43]. Crossing the BRB is an important factor affecting the efficacy of glaucoma drugs [44, 45]. Notably, various tryptophan metabolites have been demonstrated to be able to effectively cross through the blood-brain barrier (BBB) to confer their immunomodulatory effects in CNS [10, 46]. Our results also indicated that the peripheral tryptophan metabolites could go across the BRB to influence retinal AhR signaling. This addresses the critical ability of these potential drugs to cross the BBB and BRB, which may be a problem for other synthetic molecules [47–49]. In the current study, although intraperitoneal injection was used to determine the protective role of tryptophan metabolites / AhR pathway in glaucoma, therapies focus on regulating this pathway could be various. For example, investigation through oral administration is of great worth to explore in the future. Taken together, our study offers novel insights into glaucoma treatment by supplementation of AhR related tryptophan metabolites, and provides theoretical basis for future clinical transformation of this insight to restore excessive inflammatory activation mediated by disturbed tryptophan metabolism in glaucoma.

## Materials and methods

### Human serum and eyeball sample collection

For serum collection, patients with primary open angle glaucoma, primary angle closure glaucoma and cataract were recruited. Patients with cataracts were served as non-glaucoma control. Individuals with any other systemic diseases or eye diseases except ametropia were excluded. 2 mL whole blood was extracted and coagulated at room temperature, followed by centrifugated for 10 min (4 °C, 3000 g). The supernatant was collected and transferred to liquid nitrogen immediately. For eyeball collection, samples were obtained from patients who were scheduled for global enucleation because of absolute period of glaucoma with unbearable pain and from human donor eyes without any other eye diseases. The eyeball was immediately immersed in FAS eyeball fixture (Servicebio, Wuhan, China) overnight and prepared for followed experiments. Demographic and clinical characteristics of all the patients were listed in Table S1.

### Serum metabolite analysis

Serum samples were subjected to targeted metabolomics analysis by Shanghai Applied Protein Technology. Briefly, samples were prepared for HPLC-MS/MS analysis on a UPLC system (Agilent 1290 Infinity UHPLC) according to the protocol. MultiQuant was used for quantitative data processing.

### Mice

A total of 6–8 weeks old C57BL/6 J mice were obtained from Jiesijie (Shanghai, China) and were all female. Animals were housed on a 12 h light and 12 h dark cycle. All animal experiments were conducted in adherence with the guidelines prescribed by the Association for Research in Vision and Ophthalmology (ARVO). Mice were randomly divided into relevant groups for subsequent experiments.

### Retinal ischemia/reperfusion induction and treatment

Retinal ischemia/reperfusion (IR) model was inducted according to previous studies [50]. Briefly, mice were anaesthetized intraperitoneally with zolitil/dexmedetomidine mixture and were placed on a homeothermic heating blanket to maintain body temperature at 37 °C during the procedure. Pupil of the operative eye was dilated with 1% tropicamide (Santen Pharmaceutical Co.,Ltd) for 5 min. Then the operative eye was subjected to local anesthesia with 0.4% benoxinate drops (Santen Pharmaceutical Co., Ltd). 30-gauge needle cannulated to a tube infusing sterile isotonic saline (0.9% NaCl) was inserted into the anterior chamber and the needle was fixed on the back of the mouse and the heating blanket with adhesive tape. The initial IOP of mice was about 10 mmHg measured with Icare TonoLab (Icare Finland Oy, Finland), and it reached at about 90 mmHg with elevating the saline bag 120 centimeters above the mouse eye, which lasted for 60 min. ITE (10 mg/kg, Abmole, TX, USA) or CH223191 (10 mg/kg, Abmole, TX, USA) was intraperitoneally injected to mice at the same time with IR induction and was administered daily subsequently. Mice were euthanatized at day 1, day 3, and day 7 after IR injury for the following experiments, the number of each group was given in relative figure legends.

### RGC labeling and counting

Mice were euthanatized at day 7 after IR injury for RGC survival detection. Retinas were carefully separated from fixed eyeballs and dissected to retinal flat-mounts. Retinas were then permeabilized with 0.3% triton x-100 in PBS for 15 min and blocked with 5% goat serum (Boster, CA, USA) in 0.3% triton x-100 in PBS for 2 h at room temperature. After blocking, the retinas were incubated overnight at 4 °C with the primary antibody beta III tubulin (1:1000; Abcam), followed by Alexa Fluor 594 anti-mouse secondary antibody (1:200; Abbkine, CA, USA). Eight areas of each retinal flat mount were taken by confocal microscopy, the immune-positive cells were counted by ImageJ software, and the counts were averaged according to previous studies [51].

### Retinal histology and immunofluorescence

Mice were euthanatized at day 1 and 7 after IR injury and day 3 after intravitreal injection under IR for retinal histology analysis. Mice were infused with PBS to remove blood cells from retinas and perfused with 4% paraformaldehyde (PFA) for 15 min before the eyeballs were removed. Eyes were immersed in FAS eyeball fixture overnight and then embedded with paraffin and sectioned (10μm). Sections were stained with hematoxylin and eosin (H&E) for retinal thickness analysis [52]. Images were taken by microscopy. The thickness of inner plexiform layer (IPL) and retinal nerve fiber layer (RNFL) were measured at the same distance from the optic disc using ImageJ software. The cell number in ganglion cell layer (GCL) were counted in five consecutive fields from at least three different sections. Human eyeballs were sectioned in the same way introduced above and were prepared for immunofluorescence [53]. Primary antibodies used were as follows: Aryl hydrocarbon receptor (AhR; 1:200; Abclonal, Wuhan, China), Ionized calcium binding adapter molecule 1 (IBA1; 1:500; Servicebio, Wuhan, China).

### Cell culture

The BV-2 murine microglia cell line was obtained from Cellcook Biotech Co.,Ltd (Guangzhou, China) and grown in DMEM medium (Gibco) with 4.5 g/L D-Glutamine, 10 % FCS and 1 % penicillin/streptomycin in standard environment. Cells were starved 24 hours before the following experiments. For protein phosphorylation detection, subcellular fractionation and immunocytochemistry assay, ITE (1 μM) was added for 4 h following with LPS (100 ng/ml, Sigma-Aldrich) for next 4 h. For quantitative RT-PCR and western blot of inflammation cytokines, ITE (1 μM) was added for 4 hours following with LPS (100 ng/ml) for next 20 hours. CH223191 (10 μM) was pre-treated for 24 h before ITE treatment if needed.

### RNA extraction, reverse transcription, and quantitative RT-PCR

For tissue RNA extraction, mice were euthanatized at day 1 after IR injury. Total RNA from retinas was extracted using the TRIzol reagent (Invitrogen, CA, USA) according to the manufacturer’s protocol. For cell RNA extraction, total RNA from BV2 cells was extracted using the RNA extraction kit ((TaKaRa, Tokyo, Japan). Reverse transcription was performed by PrimeScript RT reagent Kit (Takara, Kyoto, Japan) following the manufacturer’s protocol. For quantification of gene expression, real time polymerase chain reaction (PCR) was performed using LightCycler 480 II (Roche, Switzerland) with UNICON qPCR SYBR Green Master Mix (Yeasen, Shanghai, China). The whole process was as follows: samples were heated to 95 °C for 10 min followed by 40 cycles of 95 °C for 5 s, and 60 °C for 20 s. PCR reaction was carried out in triplicate. Relative expression of mRNA was calculated using the 2 − Δ(ΔCT) comparative method, with each gene normalized relative to the endogenous reference gene for that sample. The primers used in the experiments were all from Tsingke (Beijing, China) and were as follows: CYP1A1 forward 5′-GTTAACCATGACCGGGAACT-3′ and reverse 5′-GTGACCTTCTCACTCAAGCG-3′, CYP1A2 forward 5′-GCAGTGGAAAGACCCCTTTG-3′ and reverse 5′- CCTTCTCGCTCTGGGTCTTG-3′, CYP1B1 forward 5′-CACCAGCCTTAGTGCAGACAG-3′ and reverse 5′-GAGGACCACGGTTTCCGTTG-3′, TNFα forward 5′-CCTCTCATGCACCACCATCA-3′ and reverse 5′- GCATTGCACCTCAGGGAAGA-3′, IL1β forward 5′-GAAATGCCACCTTTTGACAGTG-3′ and reverse 5′-TGGATGCTCTCATCAGGACAG-3′, IL6 forward 5′-GTCCGGAGAGGAGACTTCAC-3′ and reverse 5′-CTGCAAGTGCATCATCGTTGT-3′, CCL2 forward 5′-GGCGGTCAAAAAGTTTGCCT-3′ and reverse 5′- TTCTTCCGTTGAGGGACAGC-3′, iNOS forward 5′-CAAGCACCTTGGAAGAGGAG-3′ and reverse 5′-AAGGCCAAACACAGCATACC-3′, beta-actin forward 5′-CACTGTCGAGTCGCGTCC-3′ and reverse 5′- CGCAGCGATATCGTCATCCA-3′.

### Protein extraction and western blotting

Retinas and BV2 cells were isolated and lysed with RIPA (Sangon, Shanghai, China) according to the manufacturer’s protocol. Proteins were quantified using the BCA Protein Assay Kit (Beyotime, Shanghai, China). Then the proteins were separated by 10% SDS-page gels and transferred to the PVDF membrane. Primary antibodies used to incubate overnight at 4 °C were as follows: TNFα (1:1000; Abcam, Cambrige, UK), IL-1β (1:1000; Proteintech, Chicago, IL, USA), IL-6 (1:1000; Proteintech, Chicago, IL, USA), iNOS (1:1000; Affinity, USA), NFκB (1:1000; Proteintech, Chicago, IL, USA), p- NFκB (1:1000; Abcam, Cambrige, UK), ERK1/2 (1:1000; Santa Cruz Biotechnology, Santa Cruz, CA), p-ERK1/2 (1:1000; Santa Cruz Biotechnology, Santa Cruz, CA), actin (1:1000; Abmart, Shanghai, China) and beta-actin (1:1000; Bioss, Beijing, China). Blots were developed using chemiluminescence (Beyotime, Shanghai, China) and were quantified using Image J software. Band signals was normalized to actin or beta-actin.

### Subcellular fractionation and immunoblot analysis

Cytoplasm and nucleus fractions were extracted using the Nuclear and Cytoplasmic Protein Extraction Kit (Beyotime, Shanghai, China) according to the manufacturer’s protocol. Primary antibodies used were as follows: NFκB (1:1000; Proteintech, Chicago, IL, USA), AhR (1:1000; Abclonal, Wuhan, China), beta-actin (1:1000; Bioss, Beijing, China), actin (1:1000; Abmart, Shanghai, China), SP1(1:1000; Abcam, Cambrige, UK) and Histone-H3(1:1000; Proteintech, Chicago, IL, USA). Band signals was normalized to beta-actin /actin (cytoplasm) or SP1/Histone-H3 (nucleus).

### Immunocytochemistry

Cells were fixed with 4% PFA for 15 min, permeabilized and blocked with 5% goat serum (Boster, CA, USA) in 0.3% triton x-100 in PBS for 30 min at room temperature. Cells were then incubated with primary antibodies AhR (1:1000; Abclonal, Wuhan, China) or NFκB (1:1000; Proteintech, Chicago, IL, USA) overnight at 4 °C, followed by Alexa Fluor 594 anti-mouse secondary antibody (1:200; Abbkine, CA, USA). Cell nuclei were counterstained with DAPI (Invitrogen, CA, USA).

### BV2-conditioned medium preparation and intravitreal injection

Cells supernatants were filtered through a 0.45-μm syringe filter, then concentrated using the Amicon Ultra-15 centrifugal filter device with molecular weight cut-off 3-kDa (Merck Millipore, American) [54]. Concentrated conditioned medium (CCM) was then desalted twice with DPBS. The protein concentration was analyzed using the BCA Protein Assay Kit (Beyotime, Shanghai, China) and normalized to the same with DPBS for the following experiments. For intravitreal injection, two microliters of CCM or unconditioned medium was injected into the vitreous of IR and control eyes. Eyeballs were removed three days later for the follow-up experiments.

### Apoptosis detection

Retinal apoptosis was detected using the TdT-mediated dUTP nick-end labeling (TUNEL) assay kit (Servicebio, Wuhan, China) according to the manufacturer’s protocol. Digital images were captured by confocal microscopy.

### Inflammatory cytokine antibody array

Cytokines in the BV2-conditioned medium were detected using G-Series Mouse Inflammation Array 1 (RayBiotech, Inc., Norcross, GA, USA) according to the manufacturer’s protocol. In brief, 100 μl sample diluent was added to each well, blocked for 30 min and incubated for 1 h at room temperature. Fluorescence signal was visualized by a laser scanner and data were extracted using the GAL file along with the microarray analysis software.

### Statistical analysis

GraphPad Prism 8.0.2 was used for the statistical analysis. Data were presented as mean ± SEM and *p* < 0.05 was considered statistically significant. The two-sided t-test was used to perform statistical analysis between individual groups, and the one-way ANOVA followed by a Turkey’s test was used between multiple groups. Heatmap was generated based on Z-score analysis.

## Supplementary information

Table S1

checklist

supplementary figures (wb)

## Data Availability

All data included in this study are available upon request by contact with the corresponding author.

## References

[1] Quigley HA (2011). Glaucoma. Lancet (Lond, Engl).

[2] Walland MJ, Carassa RG, Goldberg I, Grehn F, Heuer DK, Khaw PT (2006). Failure of medical therapy despite normal intraocular pressure. Clin Exp Ophthalmol.

[3] Tezel G (2021). Molecular regulation of neuroinflammation in glaucoma: current knowledge and the ongoing search for new treatment targets. Prog Retin Eye Res.

[4] Baudouin C, Kolko M, Melik-Parsadaniantz S, Messmer EM (2021). Inflammation in Glaucoma: from the back to the front of the eye, and beyond. Prog Retin Eye Res.

[5] Sterling JK, Adetunji MO, Guttha S, Bargoud AR, Uyhazi KE, Ross AG (2020). GLP-1 receptor agonist NLY01 reduces retinal inflammation and neuron death secondary to ocular hypertension. Cell Rep..

[6] Guttenplan KA, Stafford BK, El-Danaf RN, Adler DI, Münch AE, Weigel MK (2020). Neurotoxic reactive astrocytes drive neuronal death after retinal injury. Cell Rep..

[7] Harder JM, Williams PA, Braine CE, Yang HS, Thomas JM, Foxworth NE (2020). Complement peptide C3a receptor 1 promotes optic nerve degeneration in DBA/2J mice. J Neuroinflammation.

[8] Krishnan A, Kocab AJ, Zacks DN, Marshak-Rothstein A, Gregory-Ksander M (2019). A small peptide antagonist of the Fas receptor inhibits neuroinflammation and prevents axon degeneration and retinal ganglion cell death in an inducible mouse model of glaucoma. J Neuroinflammation.

[9] Grotegut P, Perumal N, Kuehn S, Smit A, Dick HB, Grus FH (2020). Minocycline reduces inflammatory response and cell death in a S100B retina degeneration model. J Neuroinflammation.

[10] Rothhammer V, Mascanfroni ID, Bunse L, Takenaka MC, Kenison JE, Mayo L (2016). Type I interferons and microbial metabolites of tryptophan modulate astrocyte activity and central nervous system inflammation via the aryl hydrocarbon receptor. Nat Med.

[11] Rothhammer V, Borucki DM, Tjon EC, Takenaka MC, Chao C-C, Ardura-Fabregat A (2018). Microglial control of astrocytes in response to microbial metabolites. Nature.

[12] Lovelace MD, Varney B, Sundaram G, Lennon MJ, Lim CK, Jacobs K (2017). Recent evidence for an expanded role of the kynurenine pathway of tryptophan metabolism in neurological diseases. Neuropharmacology.

[13] Roager HM, Licht TR (2018). Microbial tryptophan catabolites in health and disease. Nat Commun.

[14] Song J, Clagett-Dame M, Peterson RE, Hahn ME, Westler WM, Sicinski RR (2002). A ligand for the aryl hydrocarbon receptor isolated from lung. Proc Natl Acad Sci USA.

[15] Munn DH, Zhou M, Attwood JT, Bondarev I, Conway SJ, Marshall B (1998). Prevention of allogeneic fetal rejection by tryptophan catabolism. Science.

[16] Proietti E, Rossini S, Grohmann U, Mondanelli G (2020). Polyamines and kynurenines at the intersection of immune modulation. Trends Immunol.

[17] Nourbakhsh B, Bhargava P, Tremlett H, Hart J, Graves J, Waubant E (2018). Altered tryptophan metabolism is associated with pediatric multiple sclerosis risk and course. Ann Clin Transl Neurol.

[18] Aeinehband S, Brenner P, Ståhl S, Bhat M, Fidock MD, Khademi M (2016). Cerebrospinal fluid kynurenines in multiple sclerosis; relation to disease course and neurocognitive symptoms. Brain Behav Immun.

[19] Phillips DH (1999). Polycyclic aromatic hydrocarbons in the diet. Mutat Res.

[20] Rothhammer V, Quintana FJ (2019). The aryl hydrocarbon receptor: an environmental sensor integrating immune responses in health and disease. Nat Rev Immunol.

[21] Shinde R, McGaha TL (2018). The aryl hydrocarbon receptor: connecting immunity to the microenvironment. Trends Immunol.

[22] Agus A, Planchais J, Sokol H (2018). Gut microbiota regulation of tryptophan metabolism in health and disease. Cell Host Microbe.

[23] Mondanelli G, Coletti A, Greco FA, Pallotta MT, Orabona C, Iacono A (2020). Positive allosteric modulation of indoleamine 2,3-dioxygenase 1 restrains neuroinflammation. Proc Natl Acad Sci USA.

[24] Harari OA, Liao JK (2010). NF-κB and innate immunity in ischemic stroke. Ann N Y Acad Sci.

[25] Yang X, Zeng Q, Barış M, Tezel G (2020). Transgenic inhibition of astroglial NF-κB restrains the neuroinflammatory and neurodegenerative outcomes of experimental mouse glaucoma. J Neuroinflammation.

[26] Fiedorowicz M, Choragiewicz T, Turski WA, Kocki T, Nowakowska D, Wertejuk K (2021). Tryptophan pathway abnormalities in a murine model of hereditary glaucoma. Int J Mol Sci.

[27] Ye M, Huang J, Mou Q, Luo J, Hu Y, Lou X (2021). CD82 protects against glaucomatous axonal transport deficits via mTORC1 activation in mice. Cell Death Dis.

[28] Wang Y, Chen S, Wang J, Liu Y, Chen Y, Wen T (2021). MicroRNA-93/STAT3 signalling pathway mediates retinal microglial activation and protects retinal ganglion cells in an acute ocular hypertension model. Cell Death Dis.

[29] Chua J, Vania M, Cheung CM, Ang M, Chee SP, Yang H (2012). Expression profile of inflammatory cytokines in aqueous from glaucomatous eyes. Mol Vis.

[30] Li S, Qiu Y, Yu J, Shao M, Li Y, Cao W (2021). Association of systemic inflammation indices with visual field loss progression in patients with primary angle-closure glaucoma: potential biomarkers for 3P medical approaches. Epma j.

[31] Grycová A, Joo H, Maier V, Illés P, Vyhlídalová B, Poulíková K (2022). Targeting the aryl hydrocarbon receptor with microbial metabolite mimics alleviates experimental colitis in mice. J Med Chem.

[32] Imran M, Chalmel F, Sergent O, Evrard B, Le Mentec H, Legrand A, et al. Transcriptomic analysis in zebrafish larvae identifies iron-dependent mitochondrial dysfunction as a possible key event of NAFLD progression induced by benzo[a]pyrene/ethanol co-exposure. Cell Biol Toxicol. 2022. 10.1007/s10565-022-09706-4 35412187

[33] Tham YC, Li X, Wong TY, Quigley HA, Aung T, Cheng CY (2014). Global prevalence of glaucoma and projections of glaucoma burden through 2040: a systematic review and meta-analysis. Ophthalmology.

[34] Platten M, Nollen EAA, Röhrig UF, Fallarino F, Opitz CA (2019). Tryptophan metabolism as a common therapeutic target in cancer, neurodegeneration and beyond. Nat Rev Drug Disco.

[35] Heilman PL, Wang EW, Lewis MM, Krzyzanowski S, Capan CD, Burmeister AR (2020). Tryptophan metabolites are associated with symptoms and nigral pathology in Parkinson’s disease. Mov Disord.

[36] Pulukool SK, Bhagavatham SKS, Kannan V, Sukumar P, Dandamudi RB, Ghaisas S (2021). Elevated dimethylarginine, ATP, cytokines, metabolic remodeling involving tryptophan metabolism and potential microglial inflammation characterize primary open angle glaucoma. Sci Rep..

[37] Sun X, Dai Y, Chen Y, Yu DY, Cringle SJ, Chen J (2017). Primary angle closure glaucoma: What we know and what we don’t know. Prog Retin Eye Res.

[38] Parker A, Romano S, Ansorge R, Aboelnour A, Le Gall G, Savva GM (2022). Fecal microbiota transfer between young and aged mice reverses hallmarks of the aging gut, eye, and brain. Microbiome.

[39] Rowan S, Jiang S, Korem T, Szymanski J, Chang ML, Szelog J (2017). Involvement of a gut-retina axis in protection against dietary glycemia-induced age-related macular degeneration. Proc Natl Acad Sci USA.

[40] Tan YQ, Wang YN, Feng HY, Guo ZY, Li X, Nie XL (2022). Host/microbiota interactions-derived tryptophan metabolites modulate oxidative stress and inflammation via aryl hydrocarbon receptor signaling. Free Radic Biol Med.

[41] Wan P, Su W, Zhang Y, Li Z, Deng C, Li J (2020). LncRNA H19 initiates microglial pyroptosis and neuronal death in retinal ischemia/reperfusion injury. Cell Death Differ.

[42] Levin LA (2017). Translational pharmacology in glaucoma neuroprotection. Handb Exp Pharm.

[43] Weinreb RN, Liebmann JM, Cioffi GA, Goldberg I, Brandt JD, Johnson CA (2018). Oral memantine for the treatment of glaucoma: design and results of 2 randomized, placebo-controlled, phase 3 studies. Ophthalmology.

[44] Yadav KS, Rajpurohit R, Sharma S (2019). Glaucoma: current treatment and impact of advanced drug delivery systems. Life Sci.

[45] Shinozaki Y, Akanuma SI, Mori Y, Kubo Y, Hosoya KI (2021). Comprehensive evidence of carrier-mediated distribution of amantadine to the retina across the blood-retinal barrier in rats. Pharmaceutics.

[46] Ortega MA, Alvarez-Mon MA, García-Montero C, Fraile-Martinez O, Guijarro LG, Lahera G (2022). Gut microbiota metabolites in major depressive disorder-deep insights into their pathophysiological role and potential translational applications. Metabolites.

[47] Banks WA (2016). From blood-brain barrier to blood-brain interface: new opportunities for CNS drug delivery. Nat Rev Drug Disco.

[48] Zhou Y, Peng Z, Seven ES, Leblanc RM (2018). Crossing the blood-brain barrier with nanoparticles. J Control Release.

[49] Kubo Y, Akanuma SI, Hosoya KI (2018). Recent advances in drug and nutrient transport across the blood-retinal barrier. Expert Opin Drug Metab Toxicol.

[50] Khanh Vu TH, Chen H, Pan L, Cho KS, Doesburg D, Thee EF (2020). CD4(+) T-cell responses mediate progressive neurodegeneration in experimental ischemic retinopathy. Am J Pathol.

[51] Cuny CS, Joachim SC, Gramlich OW, Gottschling PF, Pfeiffer N, Grus FH (2010). Repeated intraocular pressure measurement in awake Lewis rats does not bias retinal ganglion cell survival. Curr Eye Res..

[52] Fouda AY, Xu Z, Shosha E, Lemtalsi T, Chen J, Toque HA (2018). Arginase 1 promotes retinal neurovascular protection from ischemia through suppression of macrophage inflammatory responses. Cell Death Dis.

[53] Chen H, Cho KS, Vu THK, Shen CH, Kaur M, Chen G (2018). Commensal microflora-induced T cell responses mediate progressive neurodegeneration in glaucoma. Nat Commun.

[54] Jha KA, Pentecost M, Lenin R, Gentry J, Klaic L, Del Mar N (2019). TSG-6 in conditioned media from adipose mesenchymal stem cells protects against visual deficits in mild traumatic brain injury model through neurovascular modulation. Stem Cell Res Ther.

