# Supplementary figures and images for: Aryl hydrocarbon receptor dependent anti-inflammation and neuroprotective effects of tryptophan metabolites on retinal ischemia/reperfusion injury
Source: Cell Death Dis. 2023 Feb 8;14(2):92. doi: 10.1038/s41419-023-05616-3 (PMC9908897; doi:10.1038/s41419-023-05616-3)

**Fig.2G**

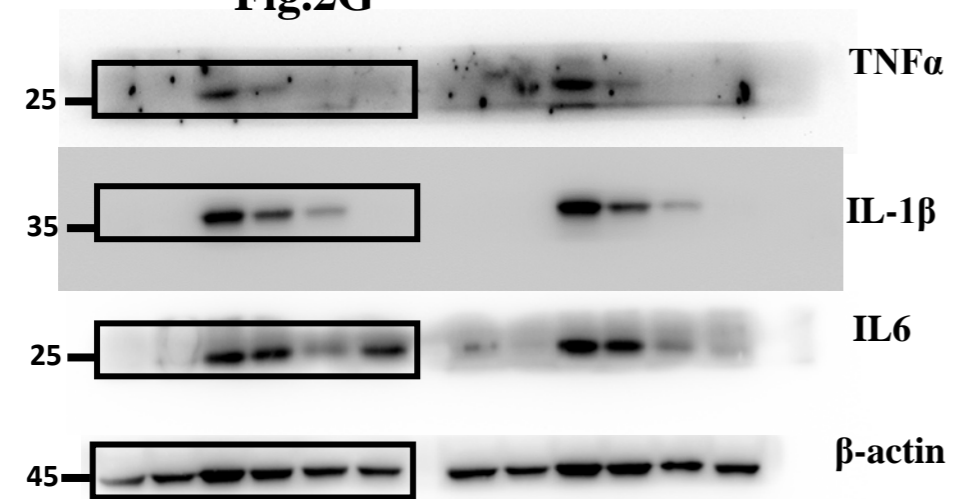

**Fig.4B**

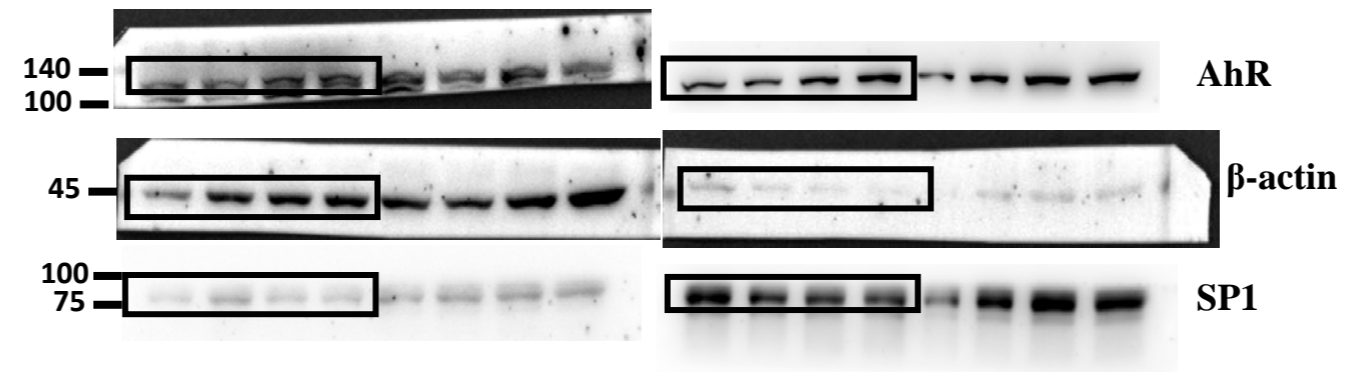

**Fig.4G**

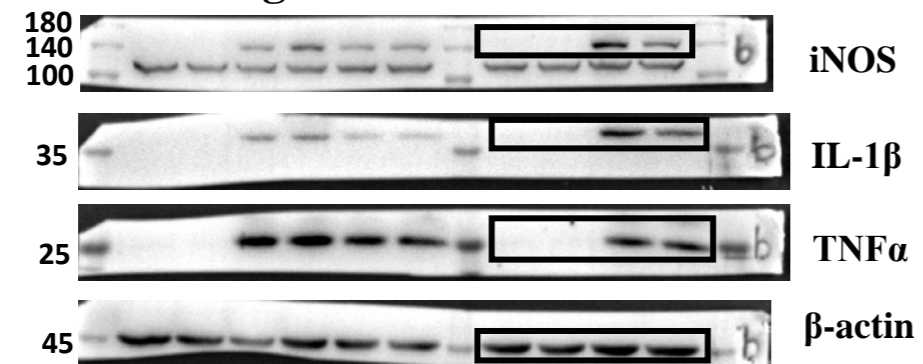

**Fig.4I**

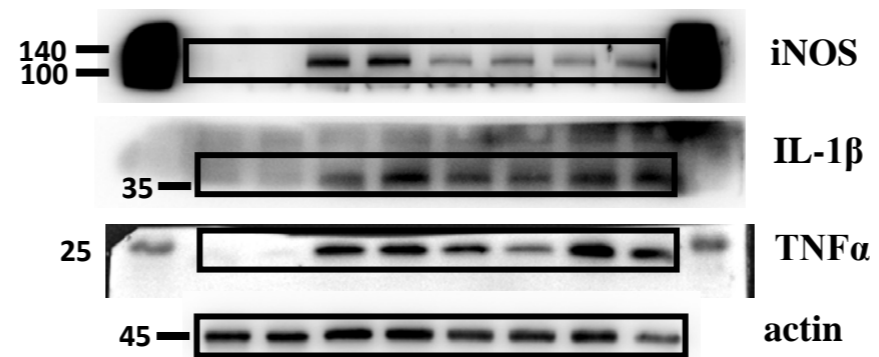

**Fig.7A**

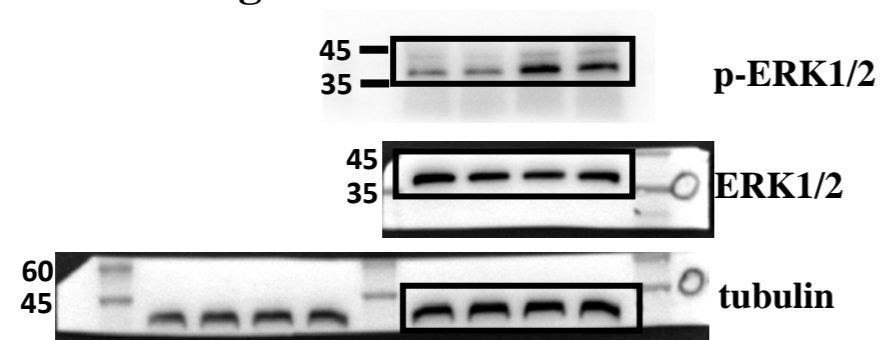

**Fig.7C**

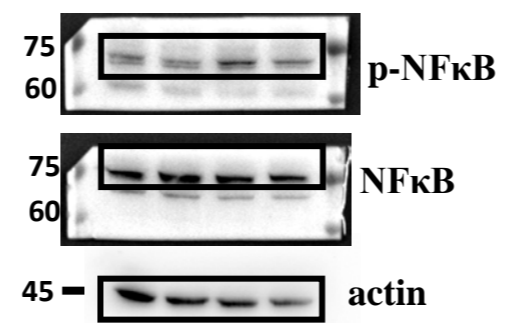

**Fig.7G**

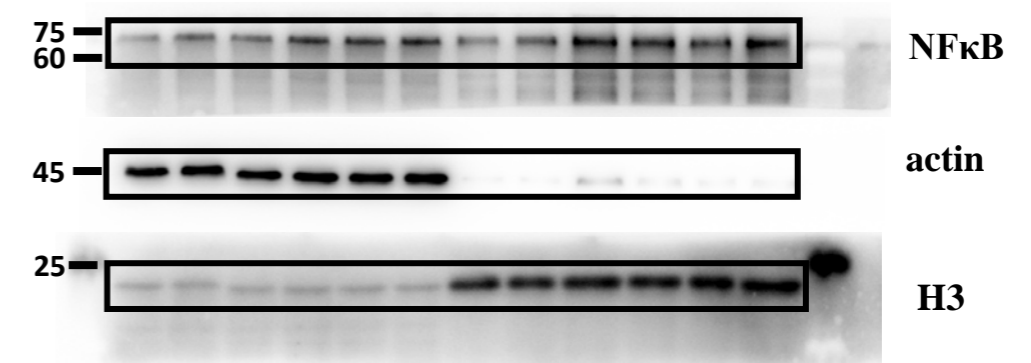

Fig.2A

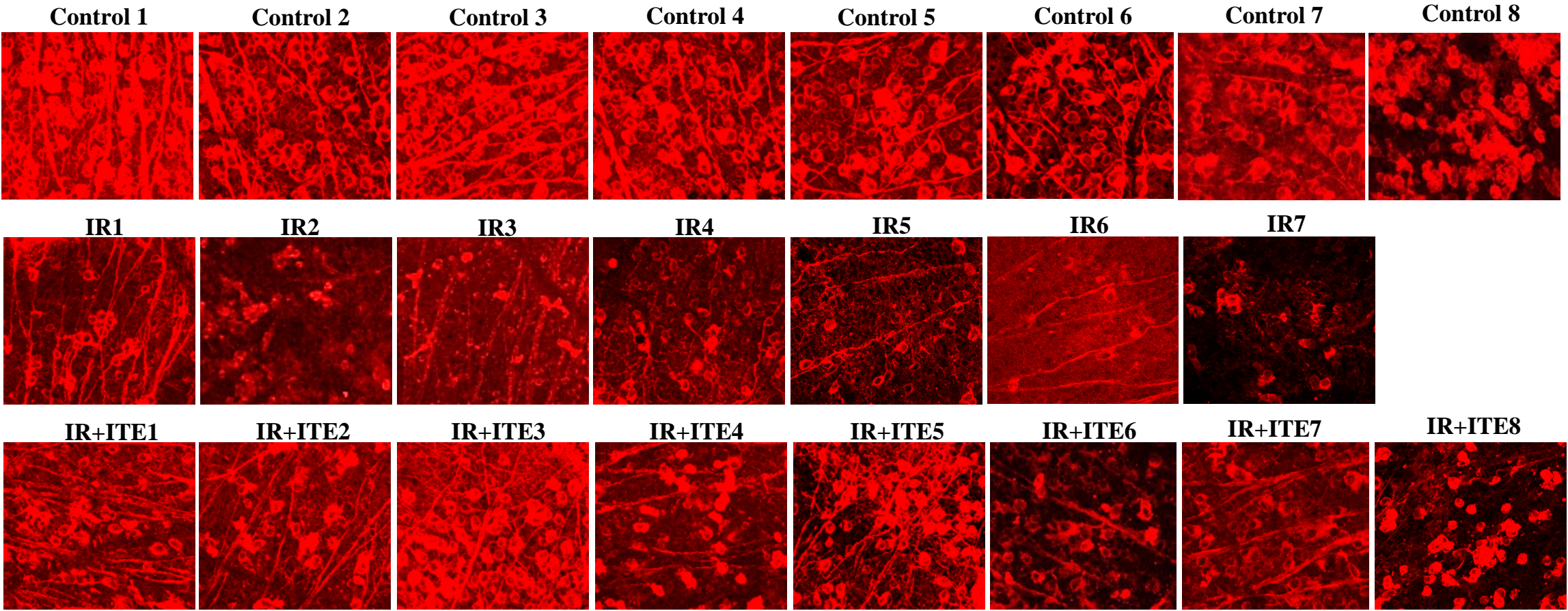

**Fig.3D**

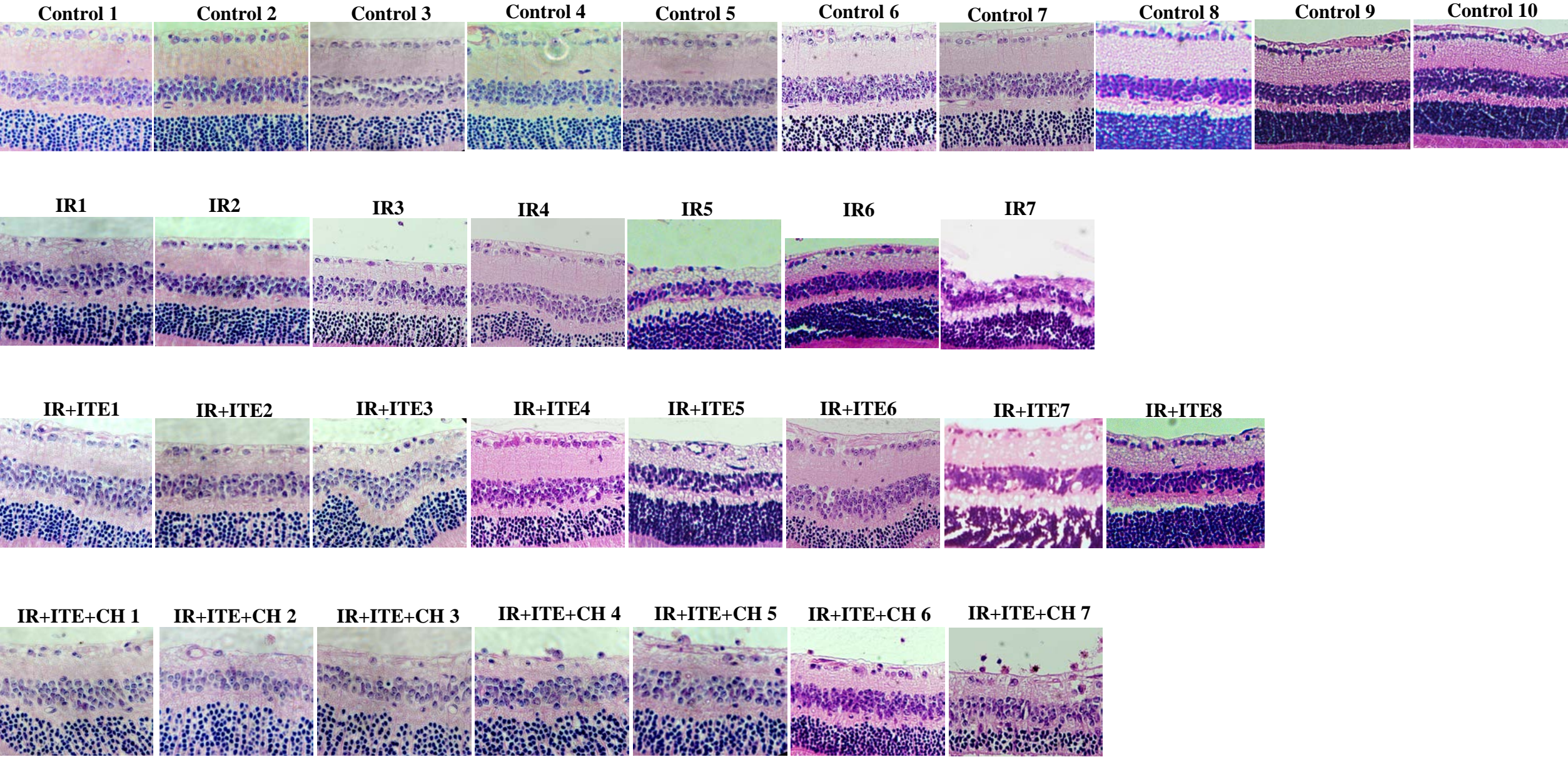

**Fig.5B-Control**

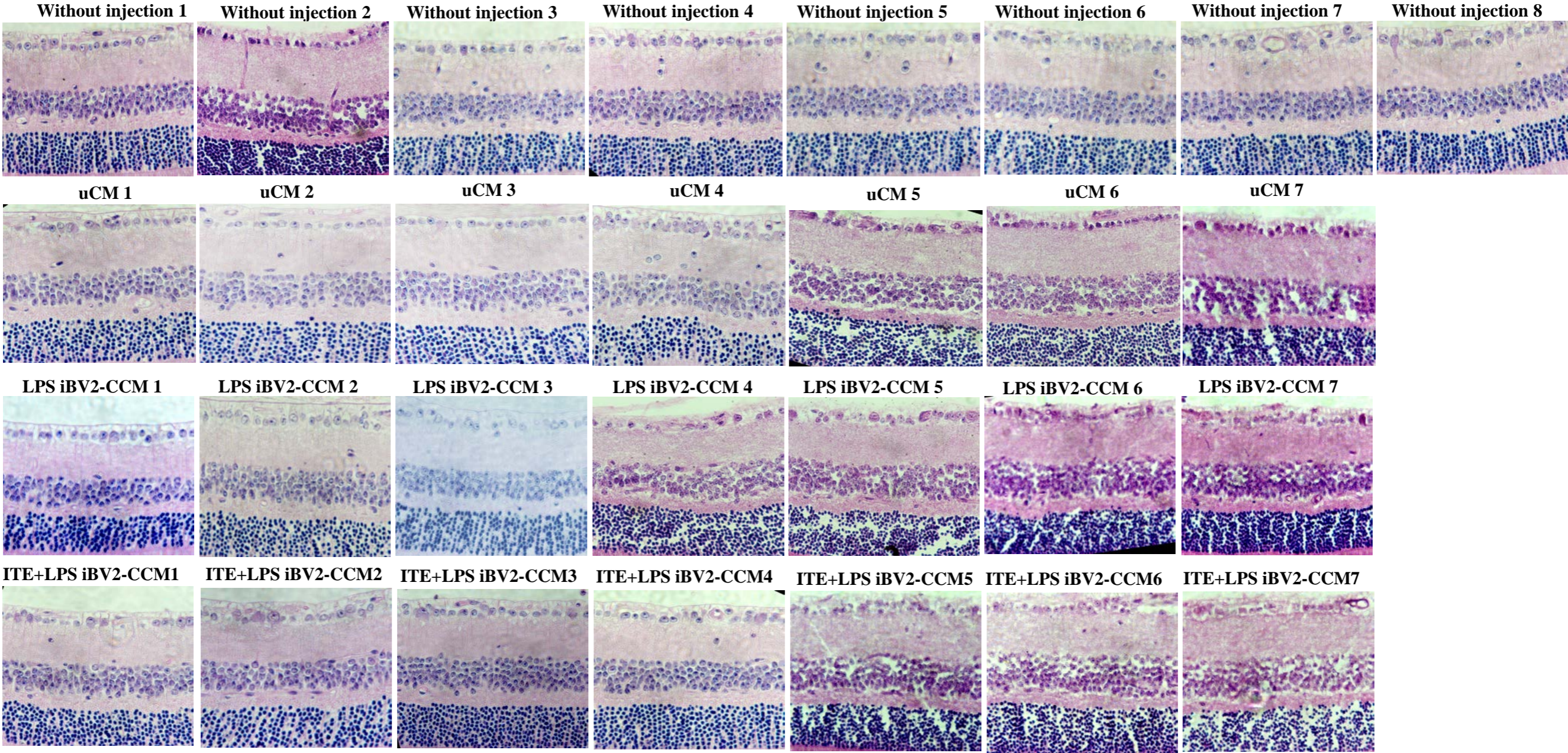

**Fig.5B-IR**

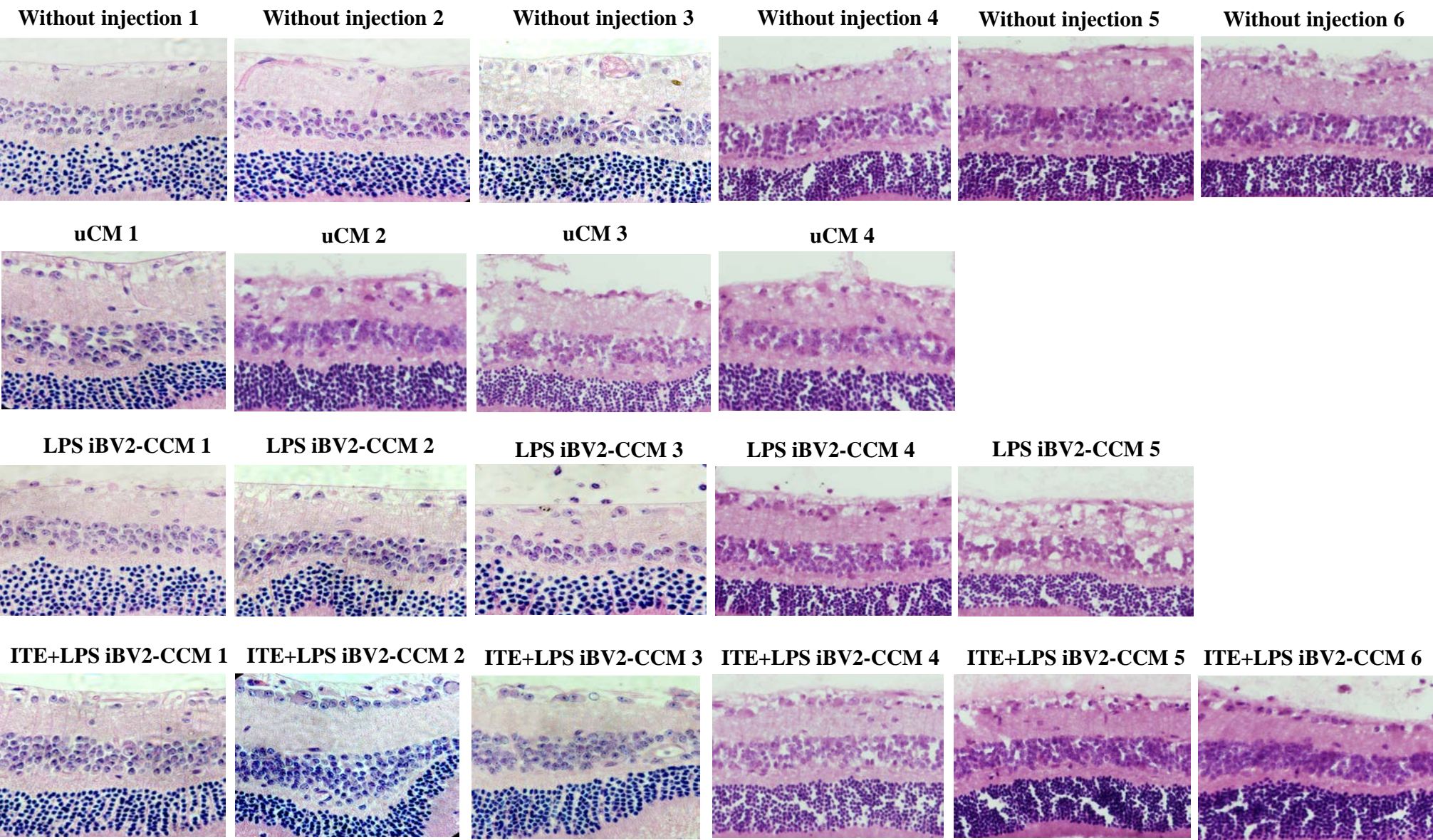

Supplement: Supplementary file 3 — supplementary figures (wb) [file 41419_2023_5616_MOESM3_ESM.pdf]
